# Network Pharmacology-Based Study on the Molecular Biological Mechanism of Action for Qingdu Decoction against Chronic Liver Injury

**DOI:** 10.1155/2021/6661667

**Published:** 2021-03-03

**Authors:** Chongyang Ma, Mengpei Zhao, Yuqiong Du, Shuang Jin, Xiaoyi Wu, Haiyan Zou, Qiuyun Zhang, Lianyin Gao

**Affiliations:** ^1^School of Traditional Chinese Medicine, Capital Medical University, Beijing 10069, China; ^2^Kaifeng Second Hospital of Traditional Chinese Medicine, Kaifeng 475000, China; ^3^Yanqing County Hospital of Traditional Chinese Medicine, Beijing 102100, China

## Abstract

**Background:**

Qingdu Decoction (QDD) is a traditional Chinese medicine formula for treating chronic liver injury (CLI). *Materials and methods*. A network pharmacology combining experimental validation was used to investigate potential mechanisms of QDD against CLI. We firstly screened the bioactive compounds with pharmacology analysis platform of the Chinese medicine system (TCMSP) and gathered the targets of QDD and CLI. Then, we constructed a compound-target network and a protein-protein interaction (PPI) network and enriched core targets in Kyoto Encyclopedia of Genes and Genomes (KEGG) signaling pathways. At last, we used a CLI rat model to confirm the effect and mechanism of QDD against CLI. Enzyme-linked immunosorbent assay (ELISA), western blot (WB), and real-time quantitative polymerase chain reaction (RT-qPCR) were used.

**Results:**

48 bioactive compounds of QDD passed the virtual screening criteria, and 53 overlapping targets were identified as core targets of QDD against CLI. A compound-CLI related target network containing 94 nodes and 263 edges was constructed. KEGG enrichment of core targets contained some pathways related to CLI, such as hepatitis B, tumor necrosis factor (TNF) signaling pathway, apoptosis, hepatitis C, interleukin-17 (IL-17) signaling pathway, and hypoxia-inducible factor (HIF)-1 signaling pathway. Three PPI clusters were identified and enriched in hepatitis B and tumor necrosis factor (TNF) signaling pathway, apoptosis and hepatitis B pathway, and peroxisome pathway, respectively. Animal experiment indicated that QDD decreased serum concentrations of alanine aminotransferase (ALT), aspartate aminotransferase (AST), endotoxin (ET), and IL-17 and increased prothrombin time activity (PTA) level. WB and RT-qPCR analyses indicated that, compared with the model group, the expression of cysteinyl aspartate specific proteinase-9 (caspase-9) protein, caspase-3 protein, B-cell lymphoma-2 associated *X* protein (Bax) mRNA, and cytochrome c (Cyt c) mRNA was inhibited and the expression of B-cell lymphoma-2 (Bcl-2) mRNA was enhanced in the QDD group.

**Conclusions:**

QDD has protective effect against CLI, which may be related to the regulation of hepatocyte apoptosis. This study provides novel insights into exploring potential biological basis and mechanisms of clinically effective formula systematically.

## 1. Introduction

Chronic liver disease (CLD) usually results from iterative liver injury, such as excessive alcohol consumption, viral hepatitis, nonalcoholic fatty liver disease, autoimmune hepatitis, primary biliary cholangitis, and primary sclerosing cholangitis, and causes approximately 2 million deaths per year worldwide [1]. Current treatments for CLD mainly lie in combined medications and artificial liver support, and liver transplant is the only recommended treatment to patients diagnosed with liver failure. Chronic liver injury (CLI), that leads to apoptosis, inflammation, matrix deposition, hyper-bilirubinemia, angiogenesis, and progressive fibrosis, is the hallmark of all types of CLDs, and targeting molecular pathways in chronic liver injury would help the development of clinical therapies to prevent or improve the prognosis of CLDs.

Traditional Chinese medicine has been around for thousands of years. There is increasing evidence that certain Chinese medicine prescriptions have beneficial effects on experimental liver injury [2]. Qingdu Decoction (QDD), a traditional Chinese medicine preparation containing *Rheum palmatum* L., *Citrus aurantium* L., *Magnolia officinalis Rehder and E.H. Wilson*, *Rehmannia glutinosa (Gaertn.) DC.*, and *Rubia cordifolia* L., has notable effects on patients with CLI. Our previous clinical studies have confirmed that the prescription has a good effect on improving clinical symptoms, protecting liver function, and reducing endotoxin absorption in CLI patients [3, 4]. And our previous experiments have shown that, in TAA-induced model rats, QDD can reduce the level of inflammatory factors and eliminate endotoxin to improve the state of liver damage [5]. We even made some efforts on the mechanisms of QDD against CLI, and a holistic understanding of its molecular mechanisms needs to be further explored.

**Figure 1:**
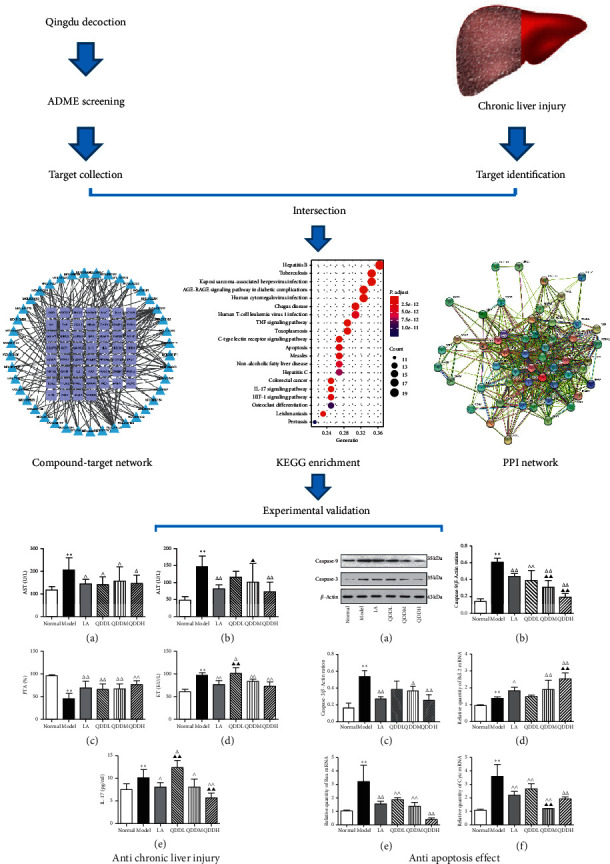
The flowchart of the whole study design.

In the present study, network pharmacology was employed to establish a compound-target network to understand the potential mechanisms of QDD against CLI. The flowchart of the whole study design is illustrated in Figure 1. Firstly, we screened bioactive compounds of QDD and mined their targets using available online databases, and then, targets related to CLI were also obtained. After intersecting these two parts of targets, targets related to both QDD and CLI were obtained and were used for further gene ontology enrichment analysis. Finally, animal experiments were used to confirm the mechanisms predicted through this in silico approach.

## 2. Materials and Methods

### 2.1. Reagent Supplies

Thioacetamide (TAA) was purchased from Keao Co., Ltd (Beijing, China). Rat ET Enzyme-linked immunoassay (ELISA) kit was from Jiancheng Inst. Biotechnology (Nanjing, China). Rat IL-17 ELISA kit was from eBioscience (Santiago, CA, USA). Primary antibodies used were rabbit caspase-9 and caspase-3 antibody (Santa cruz, Dallas, TX, USA) and mouse *β*-actin antibody (Zhongshan Jinqiao, Beijing, China). Secondary antibodies used were horseradish peroxidase-conjugated goat anti-rabbit IgG and anti-mouse IgG (Jackson, Lancaster, PA, USA). Total RNA kit was purchased from Tian Gen Biotech (Beijing, China).

### 2.2. Preparation of QDD

QDD is composed of five Chinese herbs. All the medicinal plants were provided by Beijing Tongrentang Chinese Medicine Co. Ltd. (Beijing, China). Samples were authenticated by a Prof. Zhang Qiuyun from Capital Medical University, China. Every dose of QDD contains *Rheum palmatum* L. (10 g), *Citrus aurantium* L. (10 g), *Magnolia officinalis Rehder and E.H. Wilson* (10 g), *Rehmannia glutinosa (Gaertn.) DC.* (12 g), and *Rubia cordifolia* L. (9 g). The mixture of herbal materials (51 g) was decocted with distilled water (2 × 1 h, 1 : 10 for the first extraction and 1 : 8 for the second extraction, w/v). The water extract was filtered and concentrated under reduced pressure to give the aqueous extract of QDD (34 mL). One milliliter of the extract corresponds to 1.5 g of crude drug. The decoction was stored at −20°C and heated in a 37°C water bath before gavage.

### 2.3. Animal Model and Treatment

Specific pathogen-free (SPF) grade male Wistar rats (190–210 g) were provided by Charles River Experimental Animals Technology Co. Ltd (Beijing, China). Laboratory animal use and experimental protocols have been reviewed and approved by the Ethics Committee of Capital Medical University (ethics number: AEEI-2017-109). The rats were maintained on a 12 h light/dark cycle at constant room temperature (22–25°C) and humidity (50–70%), and they have free access to standard rodent food and water.

After 7 days of adaptive feeding, 72 rats were randomly divided into six groups and each group had 12 rats: normal group, model group, lactulose (LA) group, and QDD in a low, moderate, or high dosage (QDDL, QDDM, and QDDH). The normal group was given physiological saline by gavage. The other groups were pretreated with TAA at a dose of 12 mg/kg for 8 weeks and at an increased dose of 36 mg/kg from the 9^th^ to the 12^th^ week [6]. In addition, the LA group was fed lactulose solution once a day as a positive control at a dose of 3.5 mL/kg. Each QDD group was given QDD concentrate (QDDL, QDDM, and QDDH: 5.08, 10.17, and 20.33 g/kg) daily. The whole experiment lasted 12 weeks. After intraperitoneal injection of 3% sodium pentobarbital anesthetic (30 mg/kg), all rats were sacrificed by abdominal aorta extraction.

### 2.4. Serum Biochemical Assay and Plasma Coagulation Analysis

The content of alanine transaminase (ALT) and aspartate transaminase (AST) were measured by automatic biochemical analyzer (Hitachi, Tokyo, Japan). The prothrombin time activity (PTA) was determined with coagulation by an automatic coagulation analyzer (Beckman, LAX, CA, USA).

### 2.5. Enzyme-Linked Immunoassay (ELISA) Analysis

The content of endotoxin (ET) and IL-17 in serum was measured by multiscan spectrum (Thermo, Waltham, MA, USA) using ET and IL-17 ELISA kits according to the kit's specifications.

### 2.6. Western Blot Analysis

Total protein was extracted from the liver tissues using cell lysis buffer and analyzed with bicinchoninic acid protein assay kit. Protein samples were separated on sodium dodecyl sulfate (SDS)-polyacrylamide gels and transferred onto a polyvinylidene fluoride membrane. After blocked with 5% nonfat dry milk in Tris-buffered saline containing 0.05% Tween-20 (TBST) buffer, membranes were incubated with primary antibody followed by the corresponding horseradish peroxidase-conjugated secondary antibodies. Antibodies and dilutions included the following: rabbit caspase-9 antibody (1 : 500), mouse caspase-3 antibody (1 : 500), mouse *β*-actin antibody (1 : 1,000), horseradish peroxidase-conjugated goat anti-rabbit IgG (1 : 10,000), and horseradish peroxidase-conjugated goat anti-mouse IgG (1 : 10,000).

### 2.7. Real-Time Quantitative PCR Analysis

Total RNA was extracted from liver tissues using RNA isolation kit according to the manufacturer's specification. Each sample was reverse transcribed into complementary DNA (cDNA) using the HiFi-MMLV cDNA first chain synthesis kit. Gene expression was quantified by means of the comparative Ct method (ΔΔCt), and the relative expressions were calculated by the 2^−ΔΔCt^ method.

### 2.8. Screening of Active Compounds

All compounds contained in QDD were searched for in the Traditional Chinese Medicine Systems Pharmacology Database and Analysis Platform (TCMSP; http://lsp.nwu.edu.cn/tcmsp.php), containing detailed pharmacological properties of each compound. In the present study, oral bioavailability (OB) > 30%, CACO-2 permeability > -0.4, and drug likeness (DL) > 0.18 were used for bioactive compounds screening for further analyses.

### 2.9. Identification of Targets Associated with QDD and CLI

To predict the target proteins of identified bioactive compounds in QDD, we used an approach integrated with two Chinese medicine databases, including integrative database of traditional Chinese medicine enhanced by symptom mapping (SymMap) and TCMSP [7, 8]. Comparative Toxicogenomics Database (CTD) was used for genes associated with CLI identification [9]. Only genes with Inference Score > 20 were chosen for further analysis. All these targets were transformed into gene symbols using the UniProt knowledge database (http://www.uniprot.org) with the selected species as *Homo sapiens*. Subsequently, the intersection of QDD targets and CLI targets was identified as core targets using Draw Venn Diagram online (http://bioinfogp.cnb.csic.es/tools/venny/index.html). Cytoscape 3.7.1 software was used for construction of compound-CLI related target network.

### 2.10. Construction of PPI Network of Core Targets

The protein-protein interaction (PPI) data of core targets were obtained from STRING database (https://string-db.org/). K-means algorithm was used for PPI cluster identification with default parameters supplied in STRING database. Also, PPI clusters were constructed using Cytoscape 3.7.1 software (National Resource for Network Biology, USA).

### 2.11. Pathway Enrichment Analysis of Core Targets

To identify the potential biological pathways regulated by QDD against CLI, the ClusterProfiler package of *R* 3.5.0 was adopted to conduct Kyoto Encyclopedia of Genes and Genomes (KEGG) pathway enrichment analysis of core targets [10]. Adjust *P* value < 0.05 was thought to be significant. The top 20 significantly results were further processed to produce a high-level bubble map and an enrichment map of pathways. KEGG Mapper (https://www.kegg.jp/kegg/mapper.html) was used for pathway visualization.

### 2.12. Statistical Analysis

All data were presented as means ± standard error. One-way analysis of variance (ANOVA) was performed using SPSS 19.0 (IBM Corp. Released 2010. IBM SPSS Statistics for Windows, Version 19.0. Armonk, NY, USA) to test the differences between groups. The least-significant difference (LSD) test was used for homogeneity of variance and Tamhane's T2 test for heterogeneity of variance. A value of *P*  < 0.05 was considered statistically significant.

## 3. Results

### 3.1. Therapeutic Effect of QDD against CLI

**Figure 2:**
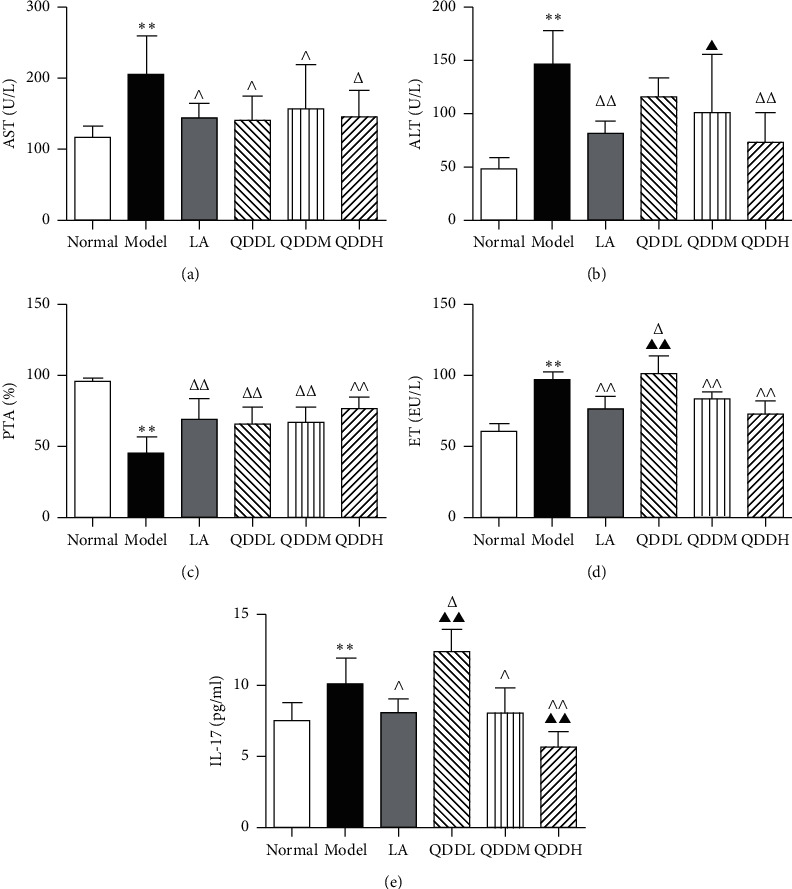
Effects of QDD on blood related indicators: (a) change in serum AST level in each group; (b) change in serum ALT level in each group; (c) change in plasma PTA level in each group; (d) change in serum ET level in each group; (e) change in serum IL-17 level in each group. Values are expressed as mean ± SEM, *n* = 6 for each group, ^*∗∗*^*P* < 0.01 vs normal group; ^Δ^*P* < 0.05 and ^ΔΔ^*P* < 0.01 vs model group; ▲*P* < 0.05 and ▲▲*P* < 0.01 vs LA group.

AST and ALT in serum were considered as important indicators of liver injury, and PTA was used to evaluate liver function. As shown in Figures 2(a)–2(c), compared to normal group, AST and ALT levels in the model group were significantly increased (*P*  < 0.01), from 115.50 U/L to 205.30 U/L and from 47.58 U/L to 145.67 U/L, respectively; and PTA was significantly decreased (*P* < 0.01), from 96.00% to 45.67%. Compared with the model group, the content of AST in the LA group and each QDD group showed different degrees of reductions (*P* < 0.05), and the content of ALT of LA and QDDH groups were significantly decreased (*P* < 0.01). PTA was significantly increased in each intervention group (*P* < 0.01). Compared with the normal group, endotoxin (ET) and interleukin (IL)-17 levels were increased in the model group (*P* < 0.01). Compared with the model group, the ET and IL-17 levels of the LA group, the QDDM group, and the QDDH group were significantly lower (*P* < 0.05 or *P* < 0.01), and these results were shown in Figures 2(d) and 2(e).

### 3.2. Screening Bioactive Compounds of QDD

To understand the potential mechanisms of QDD against CLI, a network approach was used as we previous mentioned [11]. After pharmacological properties screening, 48 bioactive compounds were identified for further analysis. As shown in Supplementary Table 1, 9 compounds were found in Dahaung, 19 compounds were found in Zhishi, 2 compounds were found in Houpo, 2 compounds were found in Dihuang, and 18 compounds were found in Qiancao.

### 3.3. Core Targets of QDD against CLI Using Network Pharmacology Approach

**Figure 3:**
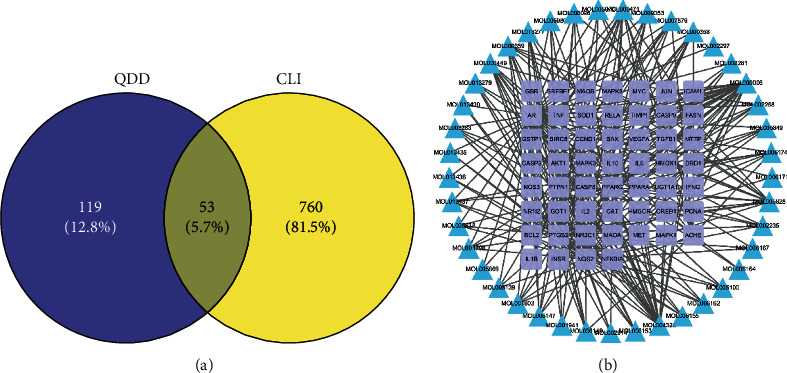
Identification of core targets of Qingdu decoction against chronic liver injury: (a) the Venn diagram of QDD targets and CLI targets; (b) compound-CLI related target network.

As shown in Supplementary Table 2 and Supplementary Table 3, a total of 813 targets associated with CLI pathogenesis and 172 targets of QDD were obtained from our integrative approach. Furthermore, 53 overlapping targets were identified via mapping targets of QDD into targets related to CLI, which were regarded as the core therapeutic targets of QDD against CLI (Figure 3(a)). Then, a compound-CLI-related target network containing 94 nodes and 263 edges was constructed (Figure 3(b)), and top 5 important compounds were identified based on degree values, including luteolin (degree: 27), naringenin (degree: 22), aloe-emodin (degree: 14), nobiletin (degree: 13), and beta-sitosterol (degree: 10).

### 3.4. KEGG Pathway Enrichment and PPI Network Construction

**Figure 4:**
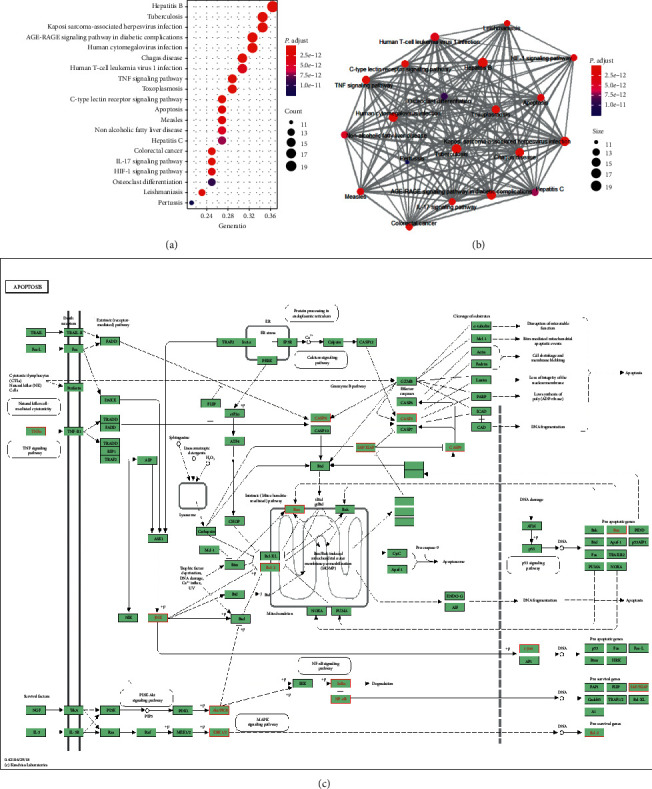
KEGG pathway enrichment analysis of core targets of Qingdu decoction against chronic liver injury: (a) bubble plot of top 20 enriched pathways; (b) enrichment map of top 20 enriched pathways; (c) modulating diagram of QDD on the apoptosis pathway. Red nodes represented core targets of QDD against CLI and green nodes represented proteins in the pathway.

**Figure 5:**
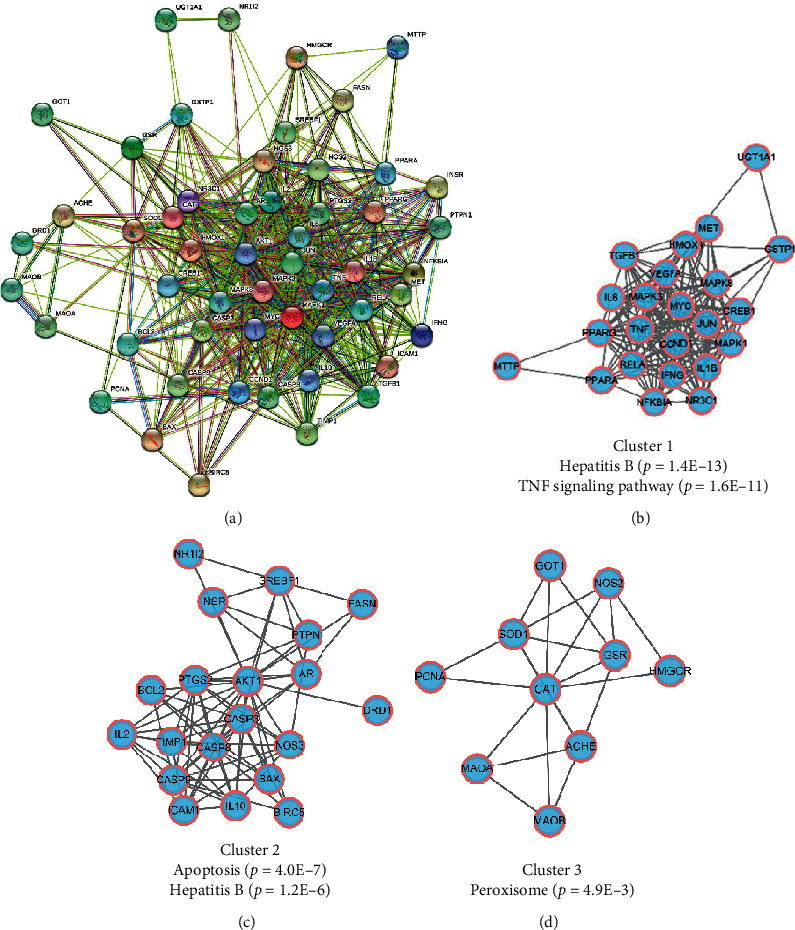
Protein-protein interaction (PPI) analysis of core targets of Qingdu decoction against chronic liver injury: (a) PPI network of core targets; (b) cluster 1 of the core PPI network; (c) cluster 2 of the core PPI network; (d) cluster 3 of the core PPI network.

To understand the related signaling pathways of core targets of QDD against CLI, KEGG pathway enrichment analysis was employed via *R* package. A total of 165 pathways were enriched and top 20 pathways ranked by adjust *P* value were visualized in a bubble plot (Figure 4(a)). In detail, some pathways related to CLI were obtained, including hepatitis B, TNF signaling pathway, apoptosis, hepatitis C, IL-17 signaling pathway, and hypoxia-inducible factor (HIF-1) signaling pathway. Enrichment map showed that top 20 pathways could be constructed as a network (Figure 4(b)). We focused on the multiple target effect of QDD on apoptosis pathway and visualized these target data in a KEGG diagram. Red nodes represented core targets of QDD against CLI (Figure 4(c)). PPI network of core targets containing 53 nodes and 620 targets was constructed with STRING website (Figure 5(a)). Also, based on topological analysis through K-means algorithm, three PPI clusters were identified (Figures 5(b)–5(d)). After KEGG pathway enrichment, we found that cluster 1 was enriched in the hepatitis B and TNF signaling pathway, cluster 2 was enriched in the apoptosis and hepatitis B pathway, and cluster 3 was enriched in the peroxisome pathway.

### 3.5. Antiapoptosis Effect of QDD against CLI

**Figure 6:**
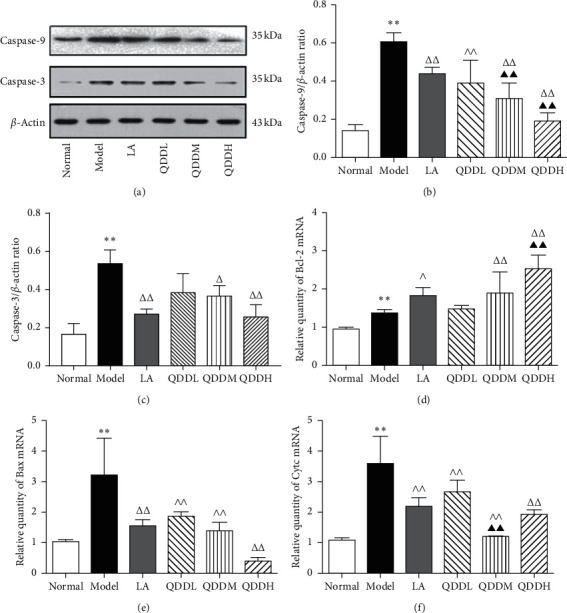
Effects of QDD on hepatocyte apoptosis. (a) Representative results of caspase-9 and caspase-3 protein; (b) the quantitative results of caspase-9 protein; (c) the quantitative results of caspase-3 protein; (d–f) quantification of Bcl-2, Bax, and Cyt c mRNA. Values are expressed as mean ± SEM, *n* = 5 for each group. ^*∗∗*^*P* < 0.01 vs normal group; ^Δ^*P* < 0.05, ^ΔΔ^*P* < 0.01 vs model group; ▲▲*P* < 0.01 vs LA group.

The above network pharmacology approach indicated a multiple target effect of QDD on apoptosis pathway, caspase-3, caspase-9, and their upstream signals including Bcl2 and Bax. Western blot analysis showed that the relative expression level of the caspase-9 protein in the model group was significantly increased (*P* < 0.01) compared with the normal group (Figures 6(a) and 6(b)). The relative expression of caspase-9 protein was decreased after treatment with QDD and LA (*P* < 0.01). Compared with the LA group, the relative expression of caspase-9 protein in the QDDM group and the QDDH group was significantly lower (*P* < 0.01). Also, the relative expression level of the caspase-3 protein in the model group was significantly increased (*P* < 0.01) compared with the normal group (Figures 6(a) and 6(c)). The relative expression of caspase-3 protein was decreased after treatment with middle (*P* < 0.05) and high dose (*P* < 0.01) of QDD. Bcl-2 and Bax are core members of the Bcl-2 family of proteins and play a crucial role in mitochondrial apoptosis [12]. Cyt *c* is a specific protein that activates caspase-9 [13]. As shown in Figures 6(c) and 6(d), the relative expression levels of Bcl-2, Bax, and Cyt c genes in the model group were significantly increased by RT-qPCR (*P* < 0.01). Compared with the model group, the relative expression levels of the Bcl-2 gene were increased in all treatment groups (*P* < 0.05 or *P* < 0.01) except the QDDL group; the relative expression levels of Bax and Cyt c genes were significantly lower in each treatment group (*P* < 0.01).

## 4. Discussion

Our previously research showed that QDD treatment inhibited CLI induced by TAA and decreased TLR4 signal activation [5]. For QDD was a multiple target preparation, it is necessary to reveal the potential mechanisms in a system level. In the present study, we used a network pharmacology approach to reveal the potential mechanisms of QDD against CLI in a system level. Compound-target network construction indicated that luteolin, naringenin, aloe-emodin, nobiletin, and beta-sitosterol were the most important compounds in the network. Literature evidence supported that all these compounds showed a very good protective effect in various CLI experimental models [14–18]. Evidence supported the ability of aloe-emodin to inhibit diverse events involved in cell apoptosis [19]. Mollugin was a potential nuclear factor kappa-B (NF*κ*B) inhibitor and decreased expression of inflammatory molecules respond to TNF signal [20, 21]. Rhein was reported to protect liver cells from methotrexate-induced injury through modulating apoptosis-related proteins, such as caspase-3 and Bcl-2 family [22]. On the contrary, some studies reported that all the above compounds could induce apoptosis in many cancer models with different doses [23–25]. Therefore, future studies should explore the differences in drug targets and involve discussion about the relationship between dose and bidirectional effect.

Core targets of QDD against CLI enriched in four categories of pathways, including disease pathways, apoptosis-related pathway, inflammatory pathway, and fibrosis-related pathway. According to KEGG enrichment analysis, these core targets were also enriched in two diseases pathways, including hepatitis B and hepatitis C. Evidence shows that hepatitis B and hepatitis C are common causes of CLI, and management of these etiologies leads to a prevention of liver inflammation and fibrosis progression [26, 27]. Both core targets of QDD against CLI and PPI cluster two were enriched in the apoptosis pathway. According to a reconstructed KEGG map, we found that core targets of QDD against CLI involved in both of the upstream and midstream of apoptosis pathway, such as TNF-*α*, Bcl-2, Bax, and Caspase-9. Our previous study has found that serum level of TNF-*α* was significantly decreased by QDD in the same CLI model [5]. Therefore, we focused on other targets in this pathway. Besides apoptosis pathway, two inflammatory pathways were obtained, including IL-17 and TNF. TNF pathway activation was the most common phenomenon in kinds of CLI animal models [28–30]. IL-17 is mainly produced from Th17 cells and upregulated in hepatitis B and C, alcoholic liver disease, and autoimmune hepatitis [31]. Literature evidence showed that inhibiting IL-17 pathway leaded to a resistance to CLI-induced liver fibrosis [27]. HIF-1 signaling pathway was also identified, and HIF-1 was confirmed as a critical regulator of profibrotic mediator production during the development of CLI-induced liver fibrosis [32]. The present study focused on the effect of QDD on apoptosis, and we designed a biological molecular experiment to reveal the potential mechanisms of QDD on the apoptosis pathway.

Similar to our previously reported study, we found protective effect of QDD against CLI. AST is mainly distributed in the mitochondria of cells, and ALT is mainly distributed in the cytoplasm of cells [33]. Therefore, the sensitivity of ALT in liver injury is higher than that of AST, and the amplitude of increase is also higher than AST [34]. On the contrary, when AST elevation exceeds ALT, it often indicates that hepatocyte injury is more serious and is a sign of aggravation of chronic disease. Plasma PTA levels mainly reflect the status of liver coagulation function and are also sensitive indicators, reflecting the degree of liver cell damage and prognosis [35].

After confirming the potential anti-CLI effect of QDD, we try to focus on the effects and mechanisms of DQQ against apoptosis. Apoptosis is one of the important causes of liver cell damage, and it is known that endotoxin is the second blow to CLI and induces chronic liver disease progression via gut-liver axis [36, 37] and directly damage liver cells and induce hepatocyte apoptosis [38, 39]. Preclinical evidence showed that LA reduced liver injury and decreased oxidative stress response, excessive inflammatory response, and fibrotic progression via downregulating endotoxin levels [40, 41]. Therefore, LA was used as positive control in the present study. By observing the ultrastructure of hepatocytes and mitochondria, it was found that the mitochondria of hepatocytes in the model group were severely swollen, accompanied by mitochondrial cristae rupture or disappearance. In severe cases, vacuolization or even rupture of the outer membrane was observed. After the intervention of QDD, the apoptosis rate of mitochondria in liver cells was significantly reduced, and the degree of mitochondrial swelling was alleviated. At the same time, it was found that QDD can effectively reduce serum endotoxin level, which was similar to LA treatment.

Inflammatory factor is another inducer of apoptosis activation. Our previous studies confirmed that previously mentioned cytokines via target prediction of QDD, such as TNF-*α*, IL-6, and IL-1*β*, were significantly induced by QDD in the same CLI model [5, 42]. The present study, we tested the serum level of IL-17 following QDD treatment, and the result indicated the potential of IL-17 as a target of QDD. Transcriptomic analysis found that luteolin had a synergistic effect on the expression level of IL-17 pathway-related genes [43], naringenin had a property of T cell-suppressive activity [44], and nobiletin was also reported to reduce IL-17 level upregulated in inflammatory model [45]. These compounds could be the biological basis of QDD on IL-17 pathway.

Under above stimulating factors, the mitochondrial membrane permeability increased, the membrane potential decreased to gradually disappear, and then, the mitochondrial morphological structure changed, leading to the release of various proapoptotic factors, which in turn leads to apoptosis. Our study found that, during the chronic liver injury, Cyt c was released and activated caspase-9, while Bax expression increased. After QDD intervention, Bax, Cyt c, caspase-9, and caspase-3 were downregulated. The experimental results also showed that the expression of antiapoptosis factor Bcl-2 mRNA was not inhibited, but increased. The role of Bcl-2 family proteins in mitochondrial apoptosis is well known [46]. As a core member of Bcl-2 family proteins, the antiapoptotic effect of Bcl-2 is mainly through the stabilization of mitochondrial membrane and regulation of intracellular Ca^2+^ concentration [47]. Bax, another core member of Bcl-2 family, can promote the release of various proapoptotic factors in mitochondria and can also form specific pores leading to the specific release of Cyt c [48]. Cyt c is a water-soluble protein located in the membrane gap of mitochondria, which is electrostatically stable in physiological conditions and bound to the inner membrane of mitochondria [49]. When stimulated by an apoptosis stimulating factor, Cyt c is released through the mitochondrial outer membrane, which promotes the binding of caspase-9 precursor to the amino terminus of apoptotic protease activating factor-1 (Apaf-1) to form an apoptotic body, further activating caspase-9 [50]. Activated caspase-9 activates the apoptotic executor caspase-3, which ultimately leads to apoptosis [51]. Back to the compound-target network, aloe-emodin, naringenin, and sitosterol were found to influence these tested targets directly, future studies should recognize the role of these compound in QDD, and pharmacokinetic parameters should be calculated in both healthy and disease individuals.

## 5. Conclusions

In conclusion, our study demonstrated that QDD can effectively reduce CLI, which is related to regulate hepatocyte apoptosis. The present study provides a new basis for the application of QDD for the treatment of CLI, which is worthy of further study.

## Data Availability

The data used to support the findings of this study are available from the corresponding author upon request.

## Abbreviations

QDD: Qingdu decoction
LA: Lactulose
ALT: Alanine aminotransferase
AST: Aspartate aminotransferase
ET: Endotoxin
IL-17: Interleukin-17
PTA: Prothrombin time activity
ELISA: Enzyme-linked immunoassay
HE: Hematoxylin-eosin
TEM: Transmission electron microscopy
WB: Western blot
qPCR: Quantitative polymerase chain reaction
Bcl-2: B-cell lymphoma-2
Bax: Bcl-2-associated *X* protein
Cyt c: Cytochrome c
Caspase-9: Cysteinyl aspartate specific proteinase-9
TNF-*α*: Tumor necrosis factor-*α*
IL-6: Interleukin-6
CLI: Chronic liver injury.

## Supplementary Materials

Supplementary Materials Supplementary Table 1. Bioactive compound information of Qingdu Decoction. Supplementary Table 2. Target information of Qingdu Decoction. Supplementary Table 3. Targets associated with chronic liver injury.
