# Supplementary material for: Network Pharmacology-Based Study on the Molecular Biological Mechanism of Action for Qingdu Decoction against Chronic Liver Injury
Source: Evid Based Complement Alternat Med. 2021 Mar 3;2021:6661667. doi: 10.1155/2021/6661667 (PMC7952185; doi:10.1155/2021/6661667)
Supplement: Supplementary Materials — Supplementary Table 1. Bioactive compound information of Qingdu Decoction. Supplementary Table 2. Target information of Qingdu Decoction. Supplementary Table 3. Targets associated with chronic liver injury. [file 6661667.f1.docx]

| Supplementary Table 1. Bioactive compound information of Qingdu Decoction | | | | | |  |
| --- | --- | --- | --- | --- | --- | --- |
| Mol ID | Molecule Name | MW | OB (%) | Caco-2 | DL | Herb |
| MOL002303 | palmidin A | 510.52 | 32.45 | -0.36 | 0.65 | Dahuang |
| MOL002297 | Daucosterol_qt | 386.73 | 35.89 | 1.35 | 0.7 | Dahuang |
| MOL002281 | Toralactone | 272.27 | 46.46 | 0.86 | 0.24 | Dahuang |
| MOL002268 | rhein | 284.23 | 47.07 | -0.2 | 0.28 | Dahuang |
| MOL002251 | Mutatochrome | 552.96 | 48.64 | 1.97 | 0.61 | Dahuang |
| MOL002235 | EUPATIN | 360.34 | 50.8 | 0.53 | 0.41 | Dahuang |
| MOL000471 | aloe-emodin | 270.25 | 83.38 | -0.12 | 0.24 | Dahuang |
| MOL000096 | (-)-catechin | 290.29 | 49.68 | -0.03 | 0.24 | Dahuang |
| MOL000358 | beta-sitosterol | 414.79 | 36.91 | 1.32 | 0.75 | Dahuang and Qiancao |
| MOL000449 | Stigmasterol | 412.77 | 43.83 | 1.44 | 0.76 | Dihuang |
| MOL000359 | sitosterol | 414.79 | 36.91 | 1.32 | 0.75 | Dihuang and Qiancao |
| MOL005980 | Neohesperidin | 302.3 | 57.44 | 0.29 | 0.27 | Houpo |
| MOL005970 | Eucalyptol | 266.36 | 60.62 | 1.1 | 0.32 | Houpo |
| MOL006174 | Xyloidone | 240.27 | 31.61 | 0.82 | 0.18 | Qiancao |
| MOL006171 | rubiprasin B | 498.87 | 35.97 | 0.99 | 0.68 | Qiancao |
| MOL006170 | Henine | 270.25 | 77.12 | -0.01 | 0.24 | Qiancao |
| MOL006167 | methyl 6-hydroxy-2,2-dimethyl-3,4-dihydrobenzo[h]chromene-5-carboxylate | 286.35 | 51.09 | 1.08 | 0.25 | Qiancao |
| MOL006164 | Pallasone | 374.62 | 43.87 | 1.04 | 0.4 | Qiancao |
| MOL006162 | Nordamnacanthal | 268.23 | 53.97 | 0.07 | 0.24 | Qiancao |
| MOL006160 | Alizarin | 240.22 | 32.67 | 0.48 | 0.19 | Qiancao |
| MOL006155 | 4-hydroxy-9,10-dioxoanthracene-2-carboxylic acid | 266.21 | 45.98 | -0.4 | 0.25 | Qiancao |
| MOL006153 | 2'-hydroxymollugin | 302.35 | 40.5 | 0.47 | 0.29 | Qiancao |
| MOL006149 | 7-hydroxy-8-methyl-4-vinyl-9,10-dihydrophenanthrene-1-carboxylic acid | 278.32 | 56.99 | 0.56 | 0.27 | Qiancao |
| MOL006147 | Alizarin-2-methylether | 254.25 | 32.81 | 0.62 | 0.21 | Qiancao |
| MOL006141 | 1,3-dihydroxy-2-hydroxymthylanthraquinone-3-O-xylosyl(1→6)-glucoside_qt | 284.28 | 71.27 | 0.05 | 0.27 | Qiancao |
| MOL006139 | 1,3-dimethoxy-2-carboxyanthraquinone | 312.29 | 102.89 | 0.32 | 0.33 | Qiancao |
| MOL005869 | daucostero_qt | 414.79 | 36.91 | 1.32 | 0.75 | Qiancao |
| MOL005638 | Mollugin | 284.33 | 42.34 | 0.87 | 0.26 | Qiancao |
| MOL003283 | (2R,3R,4S)-4-(4-hydroxy-3-methoxy-phenyl)-7-methoxy-2,3-dimethylol-tetralin-6-ol | 360.44 | 66.51 | -0.2 | 0.39 | Qiancao |
| MOL013437 | 6-Methoxy aurapten | 328.44 | 31.24 | 1.01 | 0.3 | Zhishi |
| MOL013436 | isoponcimarin | 330.41 | 63.28 | 0.5 | 0.31 | Zhishi |
| MOL013435 | poncimarin | 330.41 | 63.62 | 0.66 | 0.35 | Zhishi |
| MOL013433 | prangenin hydrate | 304.32 | 72.63 | 0.14 | 0.29 | Zhishi |
| MOL013430 | Prangenin | 286.3 | 43.6 | 0.8 | 0.29 | Zhishi |
| MOL013352 | Obacunone | 454.56 | 43.29 | 0.01 | 0.77 | Zhishi |
| MOL013279 | 5,7,4'-Trimethylapigenin | 312.34 | 39.83 | 1.01 | 0.3 | Zhishi |
| MOL013277 | Isosinensetin | 372.4 | 51.15 | 1.16 | 0.44 | Zhishi |
| MOL009053 | 4-[(2S,3R)-5-[(E)-3-hydroxyprop-1-enyl]-7-methoxy-3-methylol-2,3-dihydrobenzofuran-2-yl]-2-methoxy-phenol | 358.42 | 50.76 | 0.03 | 0.39 | Zhishi |
| MOL007879 | Tetramethoxyluteolin | 342.37 | 43.68 | 0.96 | 0.37 | Zhishi |
| MOL005849 | didymin | 286.3 | 38.55 | 0.6 | 0.24 | Zhishi |
| MOL005828 | nobiletin | 402.43 | 61.67 | 1.05 | 0.52 | Zhishi |
| MOL005100 | 5,7-dihydroxy-2-(3-hydroxy-4-methoxyphenyl)chroman-4-one | 302.3 | 47.74 | 0.28 | 0.27 | Zhishi |
| MOL004328 | naringenin | 272.27 | 59.29 | 0.28 | 0.21 | Zhishi |
| MOL002914 | Eriodyctiol (flavanone) | 288.27 | 41.35 | 0.05 | 0.24 | Zhishi |
| MOL001941 | Ammidin | 270.3 | 34.55 | 1.13 | 0.22 | Zhishi |
| MOL001803 | Sinensetin | 372.4 | 50.56 | 1.12 | 0.45 | Zhishi |
| MOL001798 | neohesperidin_qt | 302.3 | 71.17 | 0.26 | 0.27 | Zhishi |
| MOL000006 | luteolin | 286.25 | 36.16 | 0.19 | 0.25 | Zhishi |

| Supplementary Table 2. Target information of Qingdu Decoction | |
| --- | --- |
| GENE | Target name |
| NOS2 | Nitric oxide synthase, inducible |
| F2 | Prothrombin |
| ESR1 | Estrogen receptor |
| AR | Androgen receptor |
| SCN5A | Sodium channel protein type 5 subunit alpha |
| PPARG | Peroxisome proliferator-activated receptor gamma |
| F10 | Coagulation factor X |
| PTGS2 | Prostaglandin G/H synthase 2 |
| CA2 | Carbonic anhydrase 2 |
| F7 | Coagulation factor VII |
| KDR | Vascular endothelial growth factor receptor 2 |
| PTPN1 | Tyrosine-protein phosphatase non-receptor type 1 |
| TOP2A | DNA topoisomerase 2-alpha |
| ESR2 | Estrogen receptor beta |
| DPP4 | Dipeptidyl peptidase 4 |
| PPARD | Peroxisome proliferator-activated receptor delta |
| MAPK14 | Mitogen-activated protein kinase 14 |
| GSK3B | Glycogen synthase kinase-3 beta |
| HSP90AB1 | Heat shock protein HSP 90-beta |
| CDK2 | Cyclin-dependent kinase 2 |
| CHEK1 | Serine/threonine-protein kinase Chk1 |
| PRSS1 | Trypsin-1 |
| PIM1 | Serine/threonine-protein kinase pim-1 |
| CCNA2 | Cyclin-A2 |
| NCOA2 | Nuclear receptor coactivator 2 |
| CALM1 | Calmodulin-1 |
| JUN | Transcription factor AP-1 |
| PTGS1 | Prostaglandin G/H synthase 1 |
| AKR1B1 | Aldose reductase |
| PIK3CG | Phosphatidylinositol 4,5-bisphosphate 3-kinase catalytic subunit gamma isoform |
| PRKACA | cAMP-dependent protein kinase catalytic subunit alpha |
| PGR | Progesterone receptor |
| BCL2 | Apoptosis regulator Bcl-2 |
| PON1 | Serum paraoxonase/arylesterase 1 |
| MAP2 | Microtubule-associated protein 2 |
| DRD1 | D(1A) dopamine receptor |
| CHRM3 | Muscarinic acetylcholine receptor M3 |
| KCNA2 | Potassium voltage-gated channel subfamily A member 2 |
| CHRM1 | Muscarinic acetylcholine receptor M1 |
| GABRA2 | Gamma-aminobutyric acid receptor subunit alpha-2 |
| CHRM4 | Muscarinic acetylcholine receptor M4 |
| ACHE | Acetylcholinesterase |
| PDE3A | cGMP-inhibited 3',5'-cyclic phosphodiesterase A |
| HTR2A | 5-hydroxytryptamine receptor 2A |
| GABRA5 | Gamma-aminobutyric acid receptor subunit alpha-5 |
| ADRA1A | Alpha-1A adrenergic receptor |
| GABRA3 | Gamma-aminobutyric acid receptor subunit alpha-3 |
| CHRM2 | Muscarinic acetylcholine receptor M2 |
| ADRA1B | Alpha-1B adrenergic receptor |
| ADRB2 | Beta-2 adrenergic receptor |
| CHRNA2 | Neuronal acetylcholine receptor subunit alpha-2 |
| SLC6A4 | Sodium-dependent serotonin transporter |
| OPRM1 | Mu-type opioid receptor |
| NR3C1 | Glucocorticoid receptor |
| GABRA1 | Gamma-aminobutyric acid receptor subunit alpha-1 |
| CHRNA7 | Neuronal acetylcholine receptor subunit alpha-7 |
| TNF | Tumor necrosis factor |
| FASN | Fatty acid synthase |
| IL1B | Interleukin-1 beta |
| TP53 | Cellular tumor antigen p53 |
| NOS3 | Nitric oxide synthase, endothelial |
| PKIA | cAMP-dependent protein kinase inhibitor alpha |
| ABCC8 | ATP-binding cassette sub-family C member 8 |
| NCOA1 | Nuclear receptor coactivator 1 |
| KCNMA1 | Calcium-activated potassium channel subunit alpha-1 |
| ADRA2C | Alpha-2C adrenergic receptor |
| RXRA | Retinoic acid receptor RXR-alpha |
| MAOB | Amine oxidase [flavin-containing] B |
| PYGM | Glycogen phosphorylase, muscle form |
| ABAT | 4-aminobutyrate aminotransferase, mitochondrial |
| LDLR | Low-density lipoprotein receptor |
| GOT1 | Aspartate aminotransferase, cytoplasmic |
| GSR | Glutathione reductase, mitochondrial |
| ABCC1 | Multidrug resistance-associated protein 1 |
| MAPK1 | Mitogen-activated protein kinase 1 |
| MAPK3 | Mitogen-activated protein kinase 3 |
| SOD1 | Superoxide dismutase [Cu-Zn] |
| GSTP1 | Glutathione S-transferase P |
| CAT | Catalase |
| HMGCR | 3-hydroxy-3-methylglutaryl-coenzyme A reductase |
| SOAT1 | Sterol O-acyltransferase 1 |
| AKR1C1 | Aldo-keto reductase family 1 member C1 |
| PLA2G4A | Cytosolic phospholipase A2 |
| MAPK8 | Mitogen-activated protein kinase 8 |
| HNRNPH1 | Heterogeneous nuclear ribonucleoprotein H |
| MMP2 | 72 kDa type IV collagenase |
| EGFR | Epidermal growth factor receptor |
| MET | Hepatocyte growth factor receptor |
| IL6 | Interleukin-6 |
| MMP1 | Interstitial collagenase |
| IFNG | Interferon gamma |
| XDH | Xanthine dehydrogenase/oxidase |
| CDK4 | Cyclin-dependent kinase 4 |
| APP | Amyloid-beta A4 protein |
| VEGFA | Vascular endothelial growth factor A |
| TOP1 | DNA topoisomerase 1 |
| HMOX1 | Heme oxygenase 1 |
| RB1 | Retinoblastoma-associated protein |
| CASP7 | Caspase-7 |
| IL2 | Interleukin-2 |
| ADRA1D | Alpha-1D adrenergic receptor |
| NR1I2 | Nuclear receptor subfamily 1 group I member 2 |
| BACE1 | Beta-secretase 1 |
| CHRM5 | Muscarinic acetylcholine receptor M5 |
| OPRD1 | Delta-type opioid receptor |
| NR3C2 | Mineralocorticoid receptor |
| ADRB1 | Beta-1 adrenergic receptor |
| ADRA2A | Alpha-2A adrenergic receptor |
| SLC6A2 | Sodium-dependent noradrenaline transporter |
| SLC6A3 | Sodium-dependent dopamine transporter |
| PLAU | Urokinase-type plasminogen activator |
| LTA4H | Leukotriene A-4 hydrolase |
| MAOA | Amine oxidase [flavin-containing] A |
| ADH1C | Alcohol dehydrogenase 1C, gamma polypeptide |
| CTRB2 | Chymotrypsinogen B2 |
| CALM3 | Calmodulin 3 (phosphorylase kinase, delta) |
| KCNH2 | Potassium voltage-gated channel subfamily H member 2 |
| CASP3 | Caspase-3 |
| CDKN1A | Cyclin-dependent kinase inhibitor 1 |
| EIF6 | Eukaryotic translation initiation factor 6 |
| MYC | Myc proto-oncogene protein |
| PCNA | Proliferating cell nuclear antigen |
| PRKCA | Protein kinase C alpha type |
| PRKCD | Protein kinase C delta type |
| PRKCE | Protein kinase C epsilon type |
| CCNB1 | G2/mitotic-specific cyclin-B1 |
| BAX | Apoptosis regulator BAX |
| CDK1 | Cyclin-dependent kinase 1 |
| AKT1 | RAC-alpha serine/threonine-protein kinase |
| APOB | Apolipoprotein B-100 |
| BAD | Bcl2-associated agonist of cell death |
| CES1 | Liver carboxylesterase 1 |
| CYP19A1 | Aromatase |
| MTTP | Microsomal triglyceride transfer protein large subunit |
| PPARA | Peroxisome proliferator-activated receptor alpha |
| RELA | Transcription factor p65 |
| SOAT2 | Sterol O-acyltransferase 2 |
| SREBF1 | Sterol regulatory element-binding protein 1 |
| UGT1A1 | UDP-glucuronosyltransferase 1-8 |
| ADIPOQ | Adiponectin |
| PLB1 | Phospholipase B1, membrane-associated |
| BMP2 | Bone morphogenetic protein 2 |
| CD163 | Scavenger receptor cysteine-rich type 1 protein M130 |
| CREB1 | Cyclic AMP-responsive element-binding protein 1 |
| EPHB2 | Ephrin type-B receptor 2 |
| MMP9 | Matrix metalloproteinase-9 |
| RAF1 | RAF proto-oncogene serine/threonine-protein kinase |
| CASP9 | Caspase-9 |
| RASGRF2 | Ras-specific guanine nucleotide-releasing factor 2 |
| TIMP1 | Metalloproteinase inhibitor 1 |
| ADCY2 | Adenylate cyclase type 2 |
| BCL2L1 | Bcl-2-like protein 1 |
| BIRC5 | Baculoviral IAP repeat containing 5 |
| CCND1 | G1/S-specific cyclin-D1 |
| CD40LG | CD40 ligand |
| ERBB2 | Receptor tyrosine-protein kinase erbB-2 |
| ICAM1 | Intercellular adhesion molecule 1 |
| IL10 | Interleukin-10 |
| IL4 | Interleukin-4 |
| INSR | Insulin receptor |
| MCL1 | Induced myeloid leukemia cell differentiation protein Mcl-1 |
| MDM2 | E3 ubiquitin-protein ligase Mdm2 |
| NFKBIA | NF-kappa-B inhibitor alpha |
| TYR | Tyrosinase |
| XIAP | E3 ubiquitin-protein ligase XIAP |
| SLC2A4 | Solute carrier family 2, facilitated glucose transporter member 4 |
| PTGES | Prostaglandin E synthase |
| NUF2 | Kinetochore protein Nuf2 |
| CASP8 | Caspase-8 |
| TGFB1 | Transforming growth factor beta-1 |
| CRK2 | Cell division control protein 2 homolog |

| Supplementary Table 3. Targets associated with chronic liver injury. | |
| --- | --- |
| Gene Symbol | Gene ID |
| CYP7A1 | 1581 |
| GPT | 2875 |
| CYP3A4 | 1576 |
| ABCB11 | 8647 |
| FABP1 | 2168 |
| NR0B2 | 8431 |
| ALB | 213 |
| ACOX1 | 51 |
| IL6 | 3569 |
| CYP3A23-3A1 | 25642 |
| IL1A | 3552 |
| SLCO1A4 | 28250 |
| SOD2 | 6648 |
| PTGS2 | 5743 |
| BAX | 581 |
| CPT1A | 1374 |
| IFNG | 3458 |
| SLCO1A1 | 28248 |
| BCL2 | 596 |
| POR | 5447 |
| GSS | 2937 |
| CASP3 | 836 |
| ABCC3 | 8714 |
| EPHX1 | 2052 |
| IL1B | 3553 |
| GSR | 2936 |
| TXNRD1 | 7296 |
| CASP9 | 842 |
| CYP2C9 | 1559 |
| CYP2E1 | 1571 |
| PPARGC1A | 10891 |
| IL17A | 3605 |
| GPX2 | 2877 |
| STMN1 | 3925 |
| GRB14 | 2888 |
| SQSTM1 | 8878 |
| GADD45A | 1647 |
| HSDL2 | 84263 |
| CYP3A11 | 13112 |
| RIDA | 10247 |
| TXN | 7295 |
| SCD | 6319 |
| JUN | 3725 |
| RRAS2 | 22800 |
| BIRC5 | 332 |
| ABCB1A | 18671 |
| PPP1R15A | 23645 |
| FOXA1 | 3169 |
| EIF4EBP1 | 1978 |
| CASP8 | 841 |
| NQO1 | 1728 |
| ABCC2 | 1244 |
| GSTT1 | 2952 |
| ASNS | 440 |
| HMGB1 | 3146 |
| SRXN1 | 140809 |
| TRIB3 | 57761 |
| GLUD1 | 2746 |
| NDUFS1 | 4719 |
| PPARGC1B | 133522 |
| NR1I3 | 9970 |
| RBL2 | 5934 |
| PCK1 | 5105 |
| GPAT3 | 84803 |
| TXN1 | 22166 |
| GCLC | 2729 |
| DNAJC3 | 5611 |
| ACACB | 32 |
| TEX261 | 113419 |
| DECR1 | 1666 |
| MTTP | 4547 |
| GSTP1 | 2950 |
| CSTF2 | 1478 |
| DNAJC21 | 134218 |
| DUSP1 | 1843 |
| INHBE | 83729 |
| BICD2 | 23299 |
| FADS1 | 3992 |
| FABP10A | 171481 |
| BHMT | 635 |
| FGFR1OP2 | 26127 |
| TNF | 7124 |
| NREP | 9315 |
| PARP1 | 142 |
| SFN | 2810 |
| CXCL8 | 3576 |
| NFKB2 | 4791 |
| GCK | 2645 |
| IL33 | 90865 |
| SORD | 6652 |
| CDC25A | 993 |
| AKT1 | 207 |
| CYP7B1 | 9420 |
| NR1I2 | 8856 |
| CEBPB | 1051 |
| SCCPDH | 51097 |
| ALDH1A1 | 216 |
| DDIT3 | 1649 |
| SH3GL3 | 6457 |
| HTR1D | 3352 |
| HNF4A | 3172 |
| ADORA2A | 135 |
| CBR1 | 873 |
| MYC | 4609 |
| HMGB2 | 3148 |
| TXNDC16 | 57544 |
| ARPC4 | 10093 |
| ABCG5 | 64240 |
| SMARCA5 | 8467 |
| SLC22A2 | 6582 |
| HMGN1 | 3150 |
| NOS2 | 4843 |
| APOA4 | 337 |
| SOD1 | 6647 |
| CYP26A1 | 1592 |
| PKLR | 5313 |
| AQP2 | 359 |
| ACADM | 34 |
| CPPED1 | 55313 |
| LY6D | 8581 |
| ALAS1 | 211 |
| GTF2H5 | 404672 |
| ULK2 | 9706 |
| HSD11B2 | 3291 |
| RAD17 | 5884 |
| E2F5 | 1875 |
| EPN2 | 22905 |
| ITPA | 3704 |
| TK1 | 7083 |
| BMP2K | 55589 |
| HMGCR | 3156 |
| CASP2 | 835 |
| HMOX1 | 3162 |
| CXCL2 | 2920 |
| NLRP3 | 114548 |
| SLC2A2 | 6514 |
| SLC22A1 | 6580 |
| SREBF1 | 6720 |
| MMP16 | 4325 |
| PPP2R5C | 5527 |
| PRKD3 | 23683 |
| CAT | 847 |
| GCLM | 2730 |
| BCL6B | 255877 |
| COX3 | 4514 |
| SLC10A1 | 6554 |
| SERINC3 | 10955 |
| FGD4 | 121512 |
| AFP | 174 |
| GSTM1 | 2944 |
| HSD3B5 | 15496 |
| SLC25A13 | 10165 |
| TMED2 | 10959 |
| GCKR | 2646 |
| MSMO1 | 6307 |
| SQLE | 6713 |
| GABRA4 | 2557 |
| NUPR1 | 26471 |
| TIMP1 | 7076 |
| SLC30A10 | 55532 |
| BRCA1 | 672 |
| ID3 | 3399 |
| NFKBIA | 4792 |
| GPX1 | 2876 |
| HPN | 3249 |
| S100A9 | 6280 |
| AGPAT3 | 56894 |
| GAB1 | 2549 |
| HSPB6 | 126393 |
| NR3C1 | 2908 |
| PLAA | 9373 |
| FASN | 2194 |
| S100A8 | 6279 |
| TJP1 | 7082 |
| SLC3A2 | 6520 |
| ERN1 | 2081 |
| PPARG | 5468 |
| IER3 | 8870 |
| MRPS18B | 28973 |
| PPM1B | 5495 |
| CFLAR | 8837 |
| ARHGEF3 | 50650 |
| TFR2 | 7036 |
| CYBB | 1536 |
| IGFBP1 | 3484 |
| TCEA3 | 6920 |
| MET | 4233 |
| TBX21 | 30009 |
| EIF2AK3 | 9451 |
| VEGFA | 7422 |
| PDK4 | 5166 |
| TF | 7018 |
| CYP2B10 | 13088 |
| AADAT | 51166 |
| RNF19B | 127544 |
| RRM2 | 6241 |
| H2AZ1 | 3015 |
| RAMP2 | 10266 |
| IFI35 | 3430 |
| OAF | 220323 |
| RHOQ | 23433 |
| TPMT | 7172 |
| CYP2R1 | 120227 |
| CYP39A1 | 51302 |
| SNX10 | 29887 |
| TGFB1 | 7040 |
| KCNN2 | 3781 |
| FZD4 | 8322 |
| SARDH | 1757 |
| ALDH2 | 217 |
| AMIGO2 | 347902 |
| DDR1 | 780 |
| ITIH3 | 3699 |
| IL12A | 3592 |
| IGF1 | 3479 |
| PECR | 55825 |
| PIGA | 5277 |
| CD14 | 929 |
| IRS1 | 3667 |
| FOS | 2353 |
| RBBP8 | 5932 |
| MKNK2 | 2872 |
| CYP2B9 | 13094 |
| BCL2L11 | 10018 |
| CAD | 790 |
| PCNA | 5111 |
| ENTPD5 | 957 |
| KEAP1 | 9817 |
| IFIH1 | 64135 |
| LCN2 | 3934 |
| HHIPL1 | 84439 |
| GART | 2618 |
| TFF3 | 7033 |
| ALDH18A1 | 5832 |
| C1QC | 714 |
| ABCB4 | 5244 |
| NRAS | 4893 |
| FAS | 355 |
| MFN1 | 55669 |
| MAOA | 4128 |
| CYP1A2 | 1544 |
| BBOX1 | 8424 |
| MAPK13 | 5603 |
| AIFM1 | 9131 |
| ELF3 | 1999 |
| GSTT3 | 103140 |
| PSRC1 | 84722 |
| MASP1 | 5648 |
| MAPK3 | 5595 |
| NFE2L2 | 4780 |
| COL3A1 | 1281 |
| MAPK1 | 5594 |
| DTL | 51514 |
| MAP4K4 | 9448 |
| PNPLA3 | 80339 |
| IGF1R | 3480 |
| DEPP1 | 11067 |
| SRM | 6723 |
| MLXIPL | 51085 |
| LY6E | 4061 |
| UCP2 | 7351 |
| IGFBP3 | 3486 |
| CRP | 1401 |
| ABCC4 | 10257 |
| ATAD2 | 29028 |
| WIPI1 | 55062 |
| ZBTB12 | 221527 |
| GC | 2638 |
| GBE1 | 2632 |
| TNFRSF10B | 8795 |
| CCL2 | 6347 |
| ALAS2 | 212 |
| FMO2 | 2327 |
| SLC4A4 | 8671 |
| GRB10 | 2887 |
| AIM2 | 9447 |
| EME2 | 197342 |
| GFAP | 2670 |
| FYB | 23880 |
| RSPRY1 | 89970 |
| CRY1 | 1407 |
| PEX11A | 8800 |
| PLSCR1 | 5359 |
| STAT3 | 6774 |
| SCD1 | 20249 |
| MPO | 4353 |
| ACKR3 | 57007 |
| DDX58 | 23586 |
| PPARA | 5465 |
| ERO1A | 30001 |
| NELFCD | 51497 |
| RETREG2 | 79137 |
| SLC37A3 | 84255 |
| NFKB1 | 4790 |
| ACACA | 31 |
| ANG | 283 |
| TNFRSF1A | 7132 |
| CROT | 54677 |
| IFI27 | 3429 |
| CLEC2H | 94071 |
| ADRB3 | 155 |
| CCDC149 | 91050 |
| EIF2A | 83939 |
| C1QA | 712 |
| CAR1 | 12346 |
| MXD1 | 4084 |
| RAB9B | 51209 |
| SMOX | 54498 |
| EFEMP1 | 2202 |
| ADAT3 | 113179 |
| MAP1B | 4131 |
| CYP1B1 | 1545 |
| DUT | 1854 |
| GMNN | 51053 |
| FASLG | 356 |
| CSF2RB2 | 12984 |
| LBP | 3929 |
| GPR137 | 56834 |
| TXNDC12 | 51060 |
| UBALD1 | 124402 |
| DCXR | 51181 |
| GJA1 | 2697 |
| PON2 | 5445 |
| RGCC | 28984 |
| FMO5 | 2330 |
| MAPK8 | 5599 |
| CYP3A7 | 1551 |
| GSTA1 | 2938 |
| GSTA2 | 2939 |
| MX1 | 4599 |
| ACHE | 43 |
| EIF5 | 1983 |
| HSPB1 | 3315 |
| RNASE4 | 6038 |
| ROCK2 | 9475 |
| PHLDA2 | 7262 |
| PLA2G7 | 7941 |
| GBP2 | 2634 |
| DNAJC8 | 22826 |
| TUBB4B | 10383 |
| ZWINT | 11130 |
| GDAP2 | 54834 |
| ABCG8 | 64241 |
| CYP2C19 | 1557 |
| HNRNPA1 | 3178 |
| INHBB | 3625 |
| RTCB | 51493 |
| STAT1 | 6772 |
| TNFAIP8L2 | 79626 |
| ZNHIT2 | 741 |
| RORA | 6095 |
| PKNOX1 | 5316 |
| AGTR1A | 11607 |
| SLC6A6 | 6533 |
| HAL | 3034 |
| ACOT2 | 10965 |
| CLEC4N | 56620 |
| SFPQ | 6421 |
| DNAAF3 | 352909 |
| FARP2 | 9855 |
| ABRACL | 58527 |
| CIC | 23152 |
| IL12B | 3593 |
| RGN | 9104 |
| TMCO3 | 55002 |
| SLC29A1 | 2030 |
| PDIA6 | 10130 |
| SIRT1 | 23411 |
| AGGF1 | 55109 |
| MCM7 | 4176 |
| CD36 | 948 |
| IL23A | 51561 |
| LARS2 | 23395 |
| LPL | 4023 |
| MAST3 | 23031 |
| MED4 | 29079 |
| ICAM1 | 3383 |
| ACVR1B | 91 |
| PLCG1 | 5335 |
| RASD1 | 51655 |
| ALDH3A2 | 224 |
| TRNT1 | 51095 |
| RELN | 5649 |
| CASP1 | 834 |
| GPAM | 57678 |
| RELA | 5970 |
| DZIP1 | 22873 |
| NAA16 | 79612 |
| BCL3 | 602 |
| CNIH3 | 149111 |
| DES | 1674 |
| USF1 | 7391 |
| MFSD10 | 10227 |
| OBP2A | 29991 |
| TMEM258 | 746 |
| IL10 | 3586 |
| MMP12 | 4321 |
| ECEL1 | 9427 |
| HBA-A2 | 110257 |
| NFKBIZ | 64332 |
| BTBD2 | 55643 |
| CASKIN2 | 57513 |
| HAGHL | 84264 |
| LRRFIP2 | 9209 |
| TPPP | 11076 |
| MFN2 | 9927 |
| CXCL10 | 3627 |
| CYP2C70 | 226105 |
| IFIT1 | 3434 |
| UNG | 7374 |
| CBS | 875 |
| PTPN1 | 5770 |
| HAVCR1 | 26762 |
| DDX20 | 11218 |
| THUMPD1 | 55623 |
| TMEM132A | 54972 |
| TMEM184B | 25829 |
| UGT1A1 | 54658 |
| SERPINA1 | 5265 |
| B3GALNT2 | 148789 |
| CYP4A12A | 277753 |
| ACAP2 | 23527 |
| DPY19L3 | 147991 |
| HBB | 3043 |
| RCBTB1 | 55213 |
| ANXA4 | 307 |
| PRKAA1 | 5562 |
| HSD3B2 | 3284 |
| HSD17B4 | 3295 |
| COG3 | 83548 |
| MTOR | 2475 |
| FOSL2 | 2355 |
| RET | 5979 |
| SERPINA12 | 145264 |
| CDC6 | 990 |
| HERPUD1 | 9709 |
| SERPINE2 | 5270 |
| ACAT2 | 39 |
| JAK1 | 3716 |
| PBK | 55872 |
| FADD | 8772 |
| G6PC3 | 92579 |
| NOS1AP | 9722 |
| LIPC | 3990 |
| ATP1B1 | 481 |
| MRPL47 | 57129 |
| MTERF2 | 80298 |
| RAE1 | 8480 |
| GHR | 2690 |
| SLCO2B1 | 11309 |
| TLR4 | 7099 |
| IRF7 | 3665 |
| MED6 | 10001 |
| CDC42SE2 | 56990 |
| RTCA | 8634 |
| IDH1 | 3417 |
| ANKRD46 | 157567 |
| DPP8 | 54878 |
| PGS1 | 9489 |
| PWP2 | 5822 |
| RASL10B | 91608 |
| SLAMF9 | 89886 |
| DDC | 1644 |
| NDUFB7 | 4713 |
| POGLUT3 | 143888 |
| MFSD1 | 64747 |
| PHKA1 | 5255 |
| TMCO1 | 54499 |
| APC2 | 10297 |
| DDX27 | 55661 |
| MRPS22 | 56945 |
| YME1L1 | 10730 |
| PGD | 5226 |
| CHAC1 | 79094 |
| ENTPD3 | 956 |
| CYP2B6 | 1555 |
| CCR2 | 729230 |
| DPYD | 1806 |
| CLPTM1L | 81037 |
| TCAIM | 285343 |
| ADAMTS12 | 81792 |
| CLDND1 | 56650 |
| SLC35E3 | 55508 |
| USP8 | 9101 |
| NTNG1 | 22854 |
| PDCD4 | 27250 |
| PCK2 | 5106 |
| STAC2 | 342667 |
| EED | 8726 |
| CHST6 | 4166 |
| TIMM21 | 29090 |
| GGT1 | 2678 |
| ADORA1 | 134 |
| CDIP1 | 29965 |
| KLHDC2 | 23588 |
| PFDN4 | 5203 |
| RNF141 | 50862 |
| SRP68 | 6730 |
| CES2 | 8824 |
| DIS3 | 22894 |
| ADGRF1 | 266977 |
| ATF6B | 1388 |
| ELP1 | 8518 |
| IGFBP2 | 3485 |
| NAALAD2 | 10003 |
| NEU2 | 4759 |
| TUBA1A | 7846 |
| FGG | 2266 |
| ATG2B | 55102 |
| BCL9L | 283149 |
| HSBP1 | 3281 |
| RTN2 | 6253 |
| SEC22B | 9554 |
| SUGT1 | 10910 |
| UNC5A | 90249 |
| MLX | 6945 |
| CREB1 | 1385 |
| ELOVL6 | 79071 |
| DHCR24 | 1718 |
| EIF1A | 13664 |
| LYPD6 | 130574 |
| NRG4 | 145957 |
| AR | 367 |
| MTHFS | 10588 |
| NUFIP1 | 26747 |
| AGFG2 | 3268 |
| EDEM3 | 80267 |
| FOXP4 | 116113 |
| ILDR2 | 387597 |
| SENP2 | 59343 |
| STAB2 | 55576 |
| SULT2A1 | 6822 |
| VLDLR | 7436 |
| LIAS | 11019 |
| FGA | 2243 |
| LYPLAL1 | 127018 |
| NPTXR | 23467 |
| PHF5A | 84844 |
| PYCARD | 29108 |
| AATK | 9625 |
| BLOC1S2 | 282991 |
| EHHADH | 1962 |
| PDE12 | 201626 |
| PMS2 | 5395 |
| RHOBTB2 | 23221 |
| SPNS2 | 124976 |
| CPNE4 | 131034 |
| CDH1 | 999 |
| HAMP | 57817 |
| OAT | 4942 |
| SLC5A9 | 200010 |
| ADAM15 | 8751 |
| KHDRBS3 | 10656 |
| ASB13 | 79754 |
| KCTD5 | 54442 |
| MED21 | 9412 |
| PLEKHG5 | 57449 |
| RSL24D1 | 51187 |
| CKB | 1152 |
| ACAD10 | 80724 |
| EGR2 | 1959 |
| LAMTOR2 | 28956 |
| OBP3 | 259247 |
| NT5C2 | 22978 |
| PTRH2 | 51651 |
| CDC42EP5 | 148170 |
| FGF1 | 2246 |
| IL18BP | 10068 |
| BRCC3 | 79184 |
| DERL1 | 79139 |
| SESN2 | 83667 |
| GOT1 | 2805 |
| KLF4 | 9314 |
| NOL6 | 65083 |
| PHLDA1 | 22822 |
| VPS41 | 27072 |
| CYP2B2 | 361523 |
| DCAF8 | 50717 |
| TLE5 | 166 |
| TNK2 | 10188 |
| DRD1 | 1812 |
| AURKA | 6790 |
| HOXD1 | 3231 |
| ECPAS | 23392 |
| GNB5 | 10681 |
| MAP7D1 | 55700 |
| PDCD10 | 11235 |
| PRPSAP1 | 5635 |
| OSTC | 58505 |
| CCDC50 | 152137 |
| GALK2 | 2585 |
| ITSN2 | 50618 |
| PCMTD2 | 55251 |
| STRN | 6801 |
| SULT2A6 | 629219 |
| TM6SF2 | 53345 |
| GPX4 | 2879 |
| DIO1 | 1733 |
| CPLX1 | 10815 |
| GNG4 | 2786 |
| ADPGK | 83440 |
| CASP6 | 839 |
| EPC1 | 80314 |
| NRAP | 4892 |
| ALG2 | 85365 |
| CTSL | 1514 |
| CUBN | 8029 |
| MYEF2 | 50804 |
| RT1-S3 | 294228 |
| SDHD | 6392 |
| ATF6 | 22926 |
| ACLY | 47 |
| GSAP | 54103 |
| LHX6 | 26468 |
| LIN7C | 55327 |
| RNMT | 8731 |
| SLC2A13 | 114134 |
| TSPAN3 | 10099 |
| ATP2A2 | 488 |
| INSR | 3643 |
| RARA | 5914 |
| VWCE | 220001 |
| GALNS | 2588 |
| SLC2A3 | 6515 |
| GADD45G | 10912 |
| ACBD5 | 91452 |
| BLCAP | 10904 |
| DMTF1 | 9988 |
| MICALL1 | 85377 |
| N4BP2 | 55728 |
| RAB11B | 9230 |
| NDUFA8 | 4702 |
| AGO4 | 192670 |
| PKNOX2 | 63876 |
| SDAD1 | 55153 |
| SLC14A2 | 8170 |
| CHIL3 | 12655 |
| H2AJ | 55766 |
| MAN2B2 | 23324 |
| STARD5 | 80765 |
| TGFA | 7039 |
| AGPS | 8540 |
| CAB39 | 51719 |
| EPM2AIP1 | 9852 |
| IMPACT | 55364 |
| MCCC1 | 56922 |
| NADK2 | 133686 |
| PBX2 | 5089 |
| TSC22D3 | 1831 |
| LSS | 4047 |
| ACOT12 | 134526 |
| CCND1 | 595 |
| GDPD5 | 81544 |
| SLC17A2 | 10246 |
| AGTRAP | 57085 |
| DNAJC5 | 80331 |
| MAGOH | 4116 |
| NDUFA9 | 4704 |
| NIFK | 84365 |
| SAC3D1 | 29901 |
| IDH2 | 3418 |
| CRYBG1 | 202 |
| DNAJC7 | 7266 |
| LOX | 4015 |
| PPP2R5A | 5525 |
| SOX13 | 9580 |
| TOX | 9760 |
| VCAM1 | 7412 |
| CYP3A2 | 266682 |
| CEBPD | 1052 |
| MORF4L2 | 9643 |
| NSFL1C | 55968 |
| ZCCHC24 | 219654 |
| DDX10 | 1662 |
| CTNNB1 | 1499 |
| DERL3 | 91319 |
| PIK3R2 | 5296 |
| PEX19 | 5824 |
| CMTM6 | 54918 |
| HERC6 | 55008 |
| ADM | 133 |
| DYRK3 | 8444 |
| EDN2 | 1907 |
| HINT3 | 135114 |
| KIF5B | 3799 |
| PTPRU | 10076 |
| USPL1 | 10208 |
| CCDC34 | 91057 |
| RAB11FIP4 | 84440 |
| SARAF | 51669 |
| ARG1 | 383 |
| IL2 | 3558 |
| RASD2 | 23551 |
| TTPAL | 79183 |
| OAS3 | 4940 |
| MT2 | 17750 |
| CYBA | 1535 |
| FOXO1 | 2308 |
| GNL3L | 54552 |
| PLCD4 | 84812 |
| SUSD2 | 56241 |
| UBA5 | 79876 |
| AQP11 | 282679 |
| MPC1 | 51660 |
| GAB2 | 9846 |
| GOLM1 | 51280 |
| MYD88 | 4615 |
| DNAJC9 | 23234 |
| IL22 | 50616 |
| SLFN5 | 162394 |
| EGR1 | 1958 |
| H3F3B | 15081 |
| RNF6 | 6049 |
| MAOB | 4129 |
| ABI1 | 10006 |
| GRIN2D | 2906 |
| KPNA1 | 3836 |
| NPC1L1 | 29881 |
| PLEK | 5341 |
| RNF13 | 11342 |
| SCRN1 | 9805 |
| SUCLA2 | 8803 |
| UFM1 | 51569 |
| ATP6V0E2 | 155066 |
| C2CD2 | 25966 |
| MSH3 | 4437 |
| ACBD3 | 64746 |
| CYP2C55 | 72082 |
| MAFF | 23764 |
| OLFML2B | 25903 |
| TFB1M | 51106 |
| EIF2B4 | 8890 |
| TUSC3 | 7991 |
| PRXL2A | 84293 |
| TNRC18 | 84629 |
| PTPRJ | 5795 |
| SAT2 | 112483 |
| CLPX | 10845 |
| FKBP10 | 60681 |
| PI4K2B | 55300 |
| ASGR2 | 433 |
| CYP3A5 | 1577 |
| DPP3 | 10072 |
| ITPK1 | 3705 |
| MXRA7 | 439921 |
| THNSL2 | 55258 |
| CUL4A | 8451 |
| SDCBP2 | 27111 |
| SERPINE1 | 5054 |
| TAOK3 | 51347 |
| EIF2S1 | 1965 |
| RPS6 | 6194 |
| ARAF | 369 |
| CYB561 | 1534 |
| HJV | 148738 |
| SLC25A22 | 79751 |
| TSC22D2 | 9819 |
| TYK2 | 7297 |
| UPP2 | 151531 |
| CPB2 | 1361 |
| PTRH1 | 138428 |
| STIL | 6491 |
| TIMM17A | 10440 |
| ERO1B | 56605 |
| TNFAIP3 | 7128 |
| CAMP | 820 |
| CYP3A13 | 13113 |
| BCO1 | 53630 |
| DNM1 | 1759 |
| F11 | 2160 |
| MAN2B1 | 4125 |
| RCN3 | 57333 |
| TMEM98 | 26022 |
| TPST1 | 8460 |
| COX7A2 | 1347 |
| DDHD1 | 80821 |
| LACTB | 114294 |
| RFC1 | 5981 |
| NCF1 | 653361 |
| HEXA | 3073 |
| MMUT | 4594 |
| CYP4F2 | 8529 |
| G6PC | 2538 |
| GPX3 | 2878 |
| GSTM3 | 2947 |
| HEXIM1 | 10614 |
| PPP1R15B | 84919 |
| BAIAP2L1 | 55971 |
| FABP6 | 2172 |
| MYH14 | 79784 |
| NIN | 51199 |
| NIPSNAP1 | 8508 |
| PPA2 | 27068 |
| PRRG4 | 79056 |
| SORBS3 | 10174 |
| ME1 | 4199 |
| ABHD14B | 84836 |
| CYP2U1 | 113612 |
| FXYD1 | 5348 |
| PXMP4 | 11264 |
| NOS3 | 4846 |
| ABHD3 | 171586 |
| HSPB7 | 27129 |
| IPO4 | 79711 |
| MAPRE1 | 22919 |
| PANX1 | 24145 |
| SLC11A1 | 6556 |
| IER5L | 389792 |
| PRAG1 | 157285 |
| SALL1 | 6299 |
| SOCS3 | 9021 |
| TLR2 | 7097 |
| TMEM176A | 55365 |
